# Glyceraldehyde-3-phosphate dehydrogenase gene over expression correlates with poor prognosis in non small cell lung cancer patients

**DOI:** 10.1186/1476-4598-12-97

**Published:** 2013-08-29

**Authors:** Roberto Puzone, Graziana Savarino, Sandra Salvi, Maria Giovanna Dal Bello, Giulia Barletta, Carlo Genova, Erika Rijavec, Claudio Sini, Alessia Isabella Esposito, Giovanni Battista Ratto, Mauro Truini, Francesco Grossi, Ulrich Pfeffer

**Affiliations:** 1 Clinical Epidemiology Division, IRCCS AOU San Martino IST Istituto Nazionale per la Ricerca sul Cancro, Genoa, Italy; 2 Integrated Molecular Pathology Division, IRCCS AOU San Martino IST, Istituto Nazionale per la Ricerca sul Cancro, Genoa, Italy; 3 Lung Cancer Unit, IRCCS AOU San Martino IST, Istituto Nazionale per la Ricerca sul Cancro, Genoa, Italy; 4 Pathology and Cytohistology Division, IRCCS AOU San Martino IST, Istituto Nazionale per la Ricerca sul Cancro, Genoa, Italy; 5 Thoracic Surgery Division, IRCCS AOU San Martino IST, Istituto Nazionale per la Ricerca sul Cancro, Genoa, Italy

**Keywords:** Warburg effect, *GAPDH*, RQ-PCR, Gene expression microarrays, Non small cell lung cancer prognosis

## Abstract

**Background:**

Glycolysis in presence of oxygen with high glucose consumption is known to be the metabolism of choice in many tumors. In lung cancer this phenomenon is routinely exploited in diagnostic PET imaging of fluorodeoxyglucose uptake, but not much is known about the prognostic capabilities of glycolysis level assessment in resected lung tumor samples.

**Methods:**

In this retrospective study, we used real time polymerase chain reaction(RQ-PCR) to assess the expression level of the gene for Glyceraldehyde 3-phosphate dehydrogenase(GAPDH), key enzyme for glucose breakdown, in tumor samples from 82 consecutive early stages resected non small cell lung cancer(NSCLC) patients. We then compared our results in six large publicly available NSCLC microarray datasets collecting data from over 1250 total patients.

**Results:**

In our study *GAPDH* gene over expression was found to be an adverse prognostic factor in early stages NSCLC (n = 82 HR = 1.30 p = 0.050). This result was confirmed in 5 of 6 public datasets analyzed: Shedden et al. 2008: n = 442 HR = 1.54 p < 0.0001; Lee et al. 2008: n = 138 HR = 1.31 p = 0.043; Tomida et al. 2009: n = 117 HR = 1.59 p = 0.004; Roepman et al. 2009: n = 172 (TPI1 gene) HR = 1.51 p = 0.009; Okayama et al. 2012: n = 226 HR = 3.19 p < 0.0001; Botling et al. 2013: n = 196 HR = 1.00 p = 0.97). Furthermore, in the large and clinically well annotated Shedden et al. microarray dataset, *GAPDH* hazard ratio did not change whether calculated for the whole dataset or for the subgroup of adjuvant naive patients only (n = 330 HR = 1.49 p < 0.0001).

**Conclusion:**

*GAPDH* gene over expression in resected tumor samples is an adverse prognostic factor in NSCLC. Our results confirm the prognostic value of glucose metabolism assessment in NSCLC.

## Introduction

Cancer cell metabolism characterized by high glycolysis rate in presence of oxygen has been confirmed in many tumors [1]. This phenomenon, discovered by O. Warburg in 1924 [2] and once considered as the result of a “damaged” metabolism [3], has presently been found also in many rapidly multiplying non-cancerous cells, leading to an increased focus of cancer research on the specific characteristics of tumor metabolism [4].

This field of cancer research is promising. In fact the high glycolysis rate in tumors, as assessed by diagnostic positron emission tomography (PET) imaging of fluorodeoxyglucose (FDG) uptake, is also exploited in clinical practice, in the differential diagnosis of nodules of unknown origin, and, more recently, also in prognostic studies [5-7]. However, specific investigations must be performed because we can expect that tumors with different characteristics-origin, grow dynamics, etc.-have different metabolic requirements.

Diagnostic PET imaging is routinely performed in NSCLC, the most frequent histological type of lung cancer (still the leading cause of cancer death in the world [8]). There is evidence that high glucose metabolism is present in NSCLC, so a role of metabolism as prognostic factor can be hypothesized; in fact this role is actually investigated in lung cancer by the assessment of FDG uptake level [6,9,10].

New effective prognostic factors could be very useful for NSCLC patients. Presently, pathological stage of the resected tumor is the main prognostic factor used in clinical practice to select NSCLC patients to be referred for additional therapies after surgery [11], but many early staged patients actually relapse [12]. In fact, many proteins or genes, differently expressed in tumor samples from patients with different survivals, are investigated as possible prognostic biomarkers; but NSCLC is probably a very heterogeneous disease [13] and this could justify the high number of mostly non-overlapping gene lists proposed as prognostic signatures [14]. However, PET effectiveness in distinguishing NSCLC from non-tumor lung tissue suggests that genes related to glucose metabolism bear an important role in all NSCLC, regardless of tumor heterogeneity.

Among these genes, *GAPDH* has an essential role in glucose metabolism, where the corresponding enzyme converts glyceraldehydes-3-phosphate to 1,3-diphosphoglycerate with reduction of nicotinamide adenine dinucleotide (NAD+) to NADH. In fact *GAPDH* gene is expressed in all tissue, so to be classically used as housekeeping gene, but it is known to be over expressed in many tumors as compared to normal tissues, and also to be correlated with poor prognosis or tumor aggressiveness in ovarian, breast, renal, colorectal, melanoma cancer [15]. Furthermore, GAPDH protein is able to bind to RNA and DNA, supporting glycolytic and extra-glycolytic regulatory roles in cell stress, apoptosis, and metabolism [16-18]. In lung cancer, GAPDH protein is well known to be over expressed as compared to normal lung tissue [19], and *GAPDH* gene is known to be expressed at high levels as compared to the surrounding non cancerous lung biopsies [20]. However, while evidences accumulate that preoperative FDG uptake level is a prognostic factor in NSCLC, the prognostic value of *GAPDH* expression level in resected NSCLC samples is still to be assessed. In this retrospective study, we measured *GAPDH* gene expression, by RQ-PCR, on tumor samples from a group 82 resected NSCLC patients. After detecting a significant correlation of *GAPDH* with survival from our patient follow-up data, we decided to further investigate the expression of *GAPDH* gene in six large publicly available NSCLC microarray datasets, collecting data from over 1250 total NSCLC patients.

## Methods

### Study population

Our study included 82 consecutive patients, stage I-III NSCLC, who had undergone radical surgical resection at National Institute for Cancer Research, Genoa, Italy (IST) between July 2005 and March 2007. All tumors were surgically removed without microscopic residual disease. None of the patients received adjuvant radiotherapy or chemotherapy. Follow up period lasted from July 2005 to December 2010 and survival time was computed from the date of surgery. Informed written consent from the patients and approval of our institute (IST) Bioethics Board were obtained. Patient and tumor characteristics are in Table 1.

**Table 1:** IST patient’s characteristics

**Characteristics**	**Number (%)**
**Number of patients**	82
**Median Age, Years (min-max)**	69 (47–82)
**Gender**	
Female	20 (24)
Male	62 (76)
**Smoking status**	
Smokers	54 (66)
Ex-smokers	22 (27)
Never-smokers	6 (7)
**Histology**	
Adenocarcinoma	50 (61)
Squamous	28 (34)
Large cell	3 (4)
Other	1 (1)
**Stage**	
I	44 (54)
II	15 (18)
III	23 (28)
**Surgery**	
Bilobectomy	11 (13)
Lobectomy	70 (85)
Pneumonectomy	1 (1)

### Reverse transcription and RQ-PCR

RNA was isolated from paraffin-embedded tumor samples using the High Pure FFPE RNA Micro Kit (Roche Applied Science, Mannheim, Germany) with minor modifications. RNA were reverse-transcribed with SuperScript™ II RT (Invitrogen, Grand Island, NY, USA) according to the manufacturer’s instructions. Resulting cDNA was amplified by the LightCycler 480 Real Time PCR System II (Roche Applied Science). Relative gene expression levels were calculated using the Qgene software [21] featuring an efficiency corrected threshold cycle based algorithm. Beta-2-microglobulin (*B2M*) and beta-glucuronidase (*GUSB*) were used as housekeeping genes and a virtual housekeeping gene was calculated using BestKeeper software [22]. PCR primer sequences are reported in Additional file 1.

### Statistical analysis

Overall survival analysis for *GAPDH* RQ-PCR gene expression, with hazard ratio (HR) and confidence interval (CI) calculation, were performed on our patient data by using the Cox regression model. Multivariate Cox regression was performed with adjusting for tumor stage categorized in 3 classes (I – II - III). The Kaplan-Meier curve was plotted by separating patients on the median *GAPDH* gene expression level. Cumulative survivals were assessed by the Kaplan-Meier model. All calculations and plots were performed by using R 2.14(64bit) software [23]. Gene expression, survival data and sample R code are reported in Additional file 1.

### Comparison with the public microarray datasets

Six publicly available NSCLC microarrays datasets were used: Shedden et al. [24] (Sh2008) downloaded from https://array.nci.nih.gov/caarray/project/details.action?project.id=182; GSE8894, Lee et al. [25] (Le2008); GSE13213, Tomida et al. [26] (To2009); Roepman et al. [27] (Ro2009) downloaded from http://research.agendia.com (free registration is requested, but for profit usage or redistribution of data is not allowed); GSE31210, Okayama et al. [28] (Ok2012); GSE37745, Botling et al. [29] (Bo2013). Unless otherwise specified datasets were downloaded from GEO repository at http://www.ncbi.nlm.nih.gov/geo/. Full patient and tumor characteristics are in cited papers; a summary of dataset characteristics, and our patient data for comparison, is in Table 2. Datasets were chosen as being large (patients number N > 100), recent (year > =2008) and featuring adequate clinical and pathological data publicly available. For all datasets but Sh2008, we downloaded the gene expression matrix file (or equivalent files provided) with clinical and pathological data. For the Sh2008 dataset-actually the largest, and provided with high quality clinical and pathological data-we calculated the gene expression matrix from the “CEL” files provided, using standard Methods (GCRMA [30], filtering, normalization, bias corrections [31]). For this dataset two separate analysis were performed, by including (N = 442) or excluding (N = 330) the patients that had received adjuvant therapy, in order to investigate if adjuvant treatment presence could confound *GAPDH* HR results. In fact, our patients had not received adjuvant treatments but, in most of the microarray datasets, no information about adjuvant treatments was available at patient level.

**Table 2:** Summary of characteristics of the public microarray datasets compared with IST patients

**Dataset (year)**	**Patient number**	**Age median (min-max)**	**Stage I**-**II**-**III**	**NSCLS subtype**	**5**-**years cumulative survival (95% CI)**	**Microarray platform**
IST (2012)	82	69 (47–82)	44-15-23	ADK SCC other	0.54 (.44-.66)	RQ-PCR
[24] Shedden et al. (2008) all patients	442	65 (33–87)	276-96-69	ADK	0.55 (.50-.60)	Affymetrix U133a
[24] Shedden et al. (2008) adjuvant-naïve	330	65 (33–87)	230-60-40	ADK	0.60 (.55-.66)	Affyimetrix U133a
[25] Lee et al. (2008)	138	62 (13–82)	n.a.	ADK SCC	0.50 (.42-.59)	Affymetrix U133plus2
[26] Tomida et al. (2009)	117	61 (32–84)	79-13-25	ADK	0.66 (.58-.75)	Agilent 44k
[27] Roepman et al. (2009)	172	54 (22–79)	117-55-0	ADK SCC other	0.65 (.57-.74)	Agilent 44k*
[28] Okayama et al. (2012)	226	61 (30–76)	168-58-0	ADK	0.84 (.79-.89)	Affymetrix U133plus2
[29] Botling at al. (2013)	196	65 (39–84)	130-35-31	ADK SCC	0.42 (.35-.49)	Affymetrix U133plus2

*custom annotation provided.

Statistical analysis for the datasets was performed similarly as for our patient RQ-PCR data. A single *GAPDH* gene level was calculated for each patient sample as the mean level of all its probes mapped to *GAPDH*. Stage was categorized in 3 classes (I-II-III) or 2 classes (I – II + III) when patient numbers were low in higher stages. For the Le2008 dataset, relapse free survival data was used in regressions due to overall survival data unavailability. For the Ro2009 dataset, a large and clinically well annotated dataset, the probe annotation file had no *GAPDH* gene reference; we decided to use, at least in part, this dataset in the present work by analyzing the gene expression for the strictly metabolically related triosephosphate isomerase 1 (TPI1), that was found in fact highly correlated with *GAPDH* gene in all other microarray datasets (Pearson's r > 0.79 for *GAPDH*-*TPI1* expression levels). Also, patient characteristics and clinical data accuracy were diverse among the microarray datasets; so we performed the survival analysis separately for each microarray dataset, and reported the results using a forest plot [32] style comparison with our patient data, without pooling the datasets. All calculations and plots were performed by using R 2.14(64bit) software [23] and Bioconductor libraries [33].

## Results

### *GAPDH* gene expression level and correlation with survival in our patients

Patient and cancer characteristics are reported in Table 1. None of the 82 patients was lost to follow-up. During a median follow-up time of 5 years, 37 (45%) deaths were observed; cumulative survival was 89%, 73%, 65%, 56% and 54%, respectively at 1, 2, 3, 4 and 5 years. In univariate analysis age, sex, or smoking history had no correlation with survival. Only tumor stage was statistically associated with survival (stage I-II HR2.82 p = 0.019; I-III HR4.44 p = 0.0001); the median survival times were “not reached”, 2.69 and 1.80 years, for tumor stage I, II and III, respectively.

**Figure 1:**
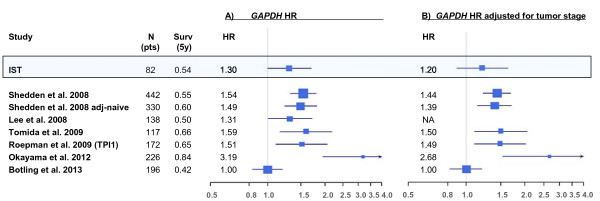
**Forest plots for *GAPDH *Hazard Ratio results in all datasets.** Forest plots style comparison for *GAPDH* Hazard Ratio (HR) Cox regression results in our patient dataset (IST) RQ-PCR measurements, and in the public microarray datasets. Confidence intervals (95%) bars and marker square sizes according to forest plot standards [32]. **A**) Comparison of HR calculated by Cox models without adjusting for tumor stage; **B**) same comparison adjusting for tumor stage in the models. Patient number (N pts) and five-years cumulative survival (Surv 5y) are also reported. A general agreement of our data with most microarray data can be observed. Botling 2013 data is an exception, in both forest plots, due to its different HR but also its low cumulative survival. Furthermore, in **B**), tumor stage adjusting has a bigger effect on IST dataset, while not much affecting any microarray dataset result.

**Figure 2:**
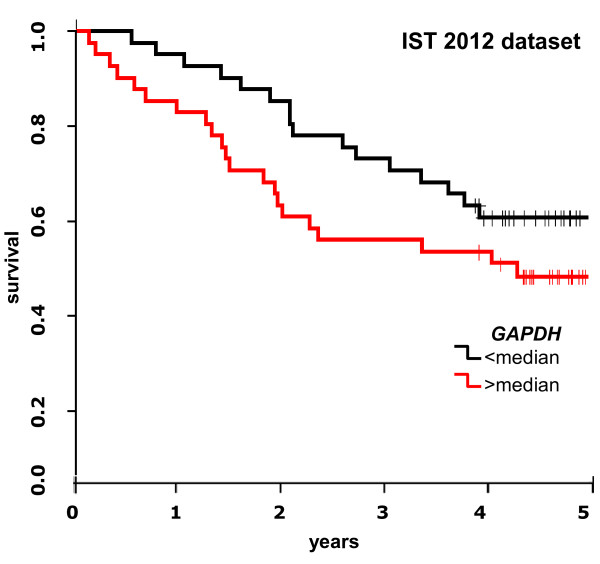
**Kaplan-Meier survival plot for our patient dataset (IST).** Kapan-Meier plots for IST dataset, where patients were divided by having *GAPDH* RQ-PCR levels higher (red line) or lower (black line) than the median level.

In univariate Cox analysis, *GAPDH* gene expression, measured by RQ-PCR, was found significantly correlated with patient survival (HR1.30; 95%CI 1.00-1.69; p = 0.050) (Figure 1A, forest plot top line). Kaplan-Meier survival plot (Figure 2), where patients are divided by *GAPDH* gene expression level being higher or lower than the median level, shows that patients with lower *GAPDH* levels had a better survival than patients with higher *GAPDH* levels.

In multivariate Cox model adjusted for stage, *GAPDH* HR value was lower than in univariate model and not significant (HR1.20; 95%CI .89-1.63; p = 0.23) (Figure 1B, forest plot top line), and only tumor stage I-III was significantly correlated with survival (stage I-II HR2.36 p = 0.069; I-III HR4.22 p = 0.0002).

### Verification in the public microarray datasets

Cox regression analysis for *GAPDH* gene expression in the microarrays datasets are summarized in the two forest plots (Figure 1A and B, before and after adjusting for tumor stage in the model, respectively), and compared with *GAPDH* results for our patient (IST) . Cumulative survivals and dataset sizes are also reported in the plots.

**Figure 3:**
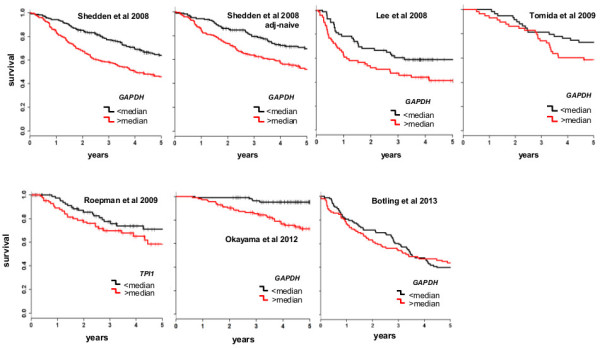
**Kaplan**-**Meier survival plots for the microarray datasets.** Kapan-Meier plots for the microarray datasets where patients were divided by having *GAPDH* probe expression levels higher (red line) or lower (black line) than the median levels. For the Roepman et al. 2009 dataset, *TPI1* probe was plotted due to *GAPDH* probe unavailability (see in Methods). It can be observed a general agreement among the datasets, and with our RQ-PCR results (Figure 2), with the exception of Botling 2013 dataset.

According to Figure 1A, the *GAPDH* HR and 95% CI values found in our patients were in good agreement with the values calculated in the microarrays datasets, with the exception of the Bo2013 dataset (HR1.00 p = 0.97). This latter also featured a five years cumulative survival lower (0.42) than most other datasets (in the range of 0.50-0.84) (Table 2) and an unusual high mortality even in lowest tumor stage patients (stage I: 130 patients, 71 deceased, 55%). In Figure 3 the Kaplan-Meier survival plots for the microarray datasets are reported, where patients are divided by *GAPDH* gene expression level being higher or lower than the median level. A substantial agreement can be observed among all Kaplan-Meier plots, and with the corresponding Kaplan-Meier plots for our patients (Figure 2).

By comparing our regression results before and after adjusting for tumor stage (respectively Figure 1A and B), it results that HR for *GAPDH* gene expression was mostly independent from stage in microarray datasets, while in our patient data (IST), after adjusting for tumor stage, HR value was decreased and not significant. In multivariate Cox proportional hazard model together with *GAPDH* gene expression, tumor stage HR values were found high (as expected) for most microarray datasets (stage: Sh2008 I-II HR2.60 p < 0.0001, I-III HR4.78 p < 0.0001;To2009 I-II + III HR2.29 p = 0.004; Ro2009-*TPI1* I-II + III HR2.13 p = 0.011; Ok2012 I-II HR1.69 p = 0.2). However, for Bo2013 dataset, tumor stage HR values were lower than in the other datasets, and not significant for stage I-II despite the high patient and event numbers (stage Bo2013 I-II HR1.28 p = 0.32; I-III HR1.88 p = 0.01).

In agreement with the rationale provided in the Methods section, the *TPI1* gene HR value and CI in Ro2009 dataset were found to be very similar to the *GAPDH* gene results in the other datasets.

Comparing the subset of the patients that did not receive adjuvant treatments, with the whole dataset in the Sh2008, we found that *GAPDH* HR was pretty unchanged (*GAPDH*: untreated patients HR1.49, whole dataset HR1.54, Figure 1A). The subset containing adjuvant treated patients only had a significantly lower cumulative survival (0.38, 95%0.30-0.50 N = 112) than the untreated patient subset (0.64, 95%.58-.70, N = 330), but *GAPDH* HR was still unchanged (adjuvant-treated *GAPDH* HR1.57, 95%1.22-2.03, p < 0.0001). Finally, in random sampling of subgroups of patients from Sh2008, we observed that *GAPDH* HR was not specifically affected when calculated in subsets featuring low cumulative survivals (Additional file 2).

## Discussion

It is well known that lung tumors present with high glycolysis level, but it is yet to demonstrate that glycolysis level, as assessed in resected NSCLC patient tumor sample, can be a prognostic factor; we think that our results gave some evidence suggesting its prognostic capabilities. In the present study we assessed the gene expression level of *GAPDH*, that has a key role in glucose breakdown; with our surprise, we found no studies specifically addressing the prognostic capabilities of *GAPDH* gene expression in resected NSCLC samples.

GAPDH protein is known to have also extra-glycolytic capabilities, being able to move to the nucleus, to support cell response to stress, and to initiate apoptosis [18]. However *GAPDH* gene is always expressed at high levels, with high glycolysis levels, in NSCLC compared to normal lung cells; so we think that our *GAPDH* prognostic results reflect an increased catalytic activity of GAPDH protein in glucose metabolism. In this sense our results are in agreement with the studies that are correlating glucose metabolism to NSCLC prognosis by using different approaches, among which FDG uptake level assessment by PET imaging of the tumor before resection. Furthermore, on the same reasoning, many studies in NSCLC are recently addressing the prognostic value of other key proteins or gene involved in glucose metabolism, e.g. GLUT1, HK2 [10]. In fact, it is still unknown which aspects of glycolysis have strong prognostic value in NSCLC, but many available evidences, including our present study results, support that the level of glycolysis has indeed prognostic value.

In our study we measured RQ-PCR *GAPDH* gene expression levels in the resected tumors from 82 patients of our hospital and found a significant correlation with their prognosis. Then we decided to verify this correlation in the largest NSCLC public microarray datasets, and we found a confirmation of our result. We showed all results in forest plot style, for an individual comparison. In fact, not all the available public data feature the same accuracy; especially some datasets, e.g. Sh2008, are better annotated so to be used as a reference in many studies. Among the confirmations coming from the microarray datasets, we think that the Sh2008 data gave a strong support to our results.

Our results for *GAPDH* also agree with the findings of a very recent paper from Wang et al. [34] in which the authors show the prognostic value of some genes correlated with *GAPDH* (GACC genes) together with *GAPDH* itself; Sh2008 was used as verification dataset. Authors don't show the prognostic performance of *GAPDH* alone; however, our results, confirmed on a large number of public datasets including Sh2008, suggest that large part of the prognostic performances shown in Sh2008 have to be attributed to *GAPDH* alone.

In the forest plots we showed the Ro2009 dataset results too, by plotting its *TPI1* gene levels instead of the unavailable *GAPDH* ones. Actually this substitution was based on the strict metabolic relation between the two catalytic proteins – however the high correlation of the two genes was verified in the other datasets, and is confirmed by other authors too [34]. So, Ro2009 results for *TPI1*, very similar to *GAPDH* results in the other datasets, can further support that the prognostic capabilities of *GAPDH* in NSCLC reflect the role of the corresponding enzyme in glucose metabolism.

However one dataset (Bo2013) had a null result for *GAPDH* correlation with prognosis (HR = 1.0); this dataset was also featuring some characteristics different from all the other ones: i) a low cumulative survival, also at low tumor stages, and ii) a low tumor stage HR and significance, despite the high patient and event numbers. We have no data supporting a correlation of these characteristics with a strong decrease of HR values for *GAPDH*, so we can only conclude that the Bo2013 dataset is different from the other datasets from more than a single point of view.

*GAPDH* HR was not affected when selecting only patients that had not received any adjuvant therapy; we performed this comparison in the Sh2008 dataset. This result was helpful for our data analysis; in fact our patients had not received radiotherapy or chemotherapy, but in most microarray datasets the information, whether adjuvant treatments had been performed or not, was not available at patient level. Actually, adjuvant treatment presence could confound a survival analysis because there is-finally- evidence that it can increase survival also in lower stages patient [12]. Furthermore, clinicians select patients with presumed poor prognosis to be referred for adjuvant therapies-in fact patient selection is one of the main reasons why retrospective studies cannot address adjuvant treatment effectiveness; this selection was resulting in the low cumulative survival found in Sh2008 adjuvant treated patient only subset. However, we observed that this selection probably did not much influence *GAPDH* HR value (Additional file 2). So, *GAPDH* HR insensitivity to the presence of adjuvant treatments suggests that *GAPDH* is still a prognostic factor in adjuvant treated patients, but is not promising as predictive factor of adjuvant effectiveness, as performed in Sh2008 patients.

However, in more recent years, some anti-tumor drugs under investigation are involving tumor metabolism, e.g. by reducing glucose availability as metformin [35], or by directly targeting glycolysis proteins [36]; our results suggest that in clinical investigations on these drugs, *GAPDH* levels in resected NSCLC samples should be investigated as possible predictor of treatment effectiveness.

From the clinical point of view the *GAPDH* HR value found in our patients is interesting; however after tumor stage adjusting, significance was lost, pointing out that *GAPDH* gene expression had some correlation with tumor stage. Indeed, adjusting for tumor stage in the regression model had small effect on HR calculation in microarray datasets, suggesting that our patient number was simply critically too low to overpass the significance level for HR after adjusting for stage, but that *GAPDH* HR is for large part independent from stage. It will be therefore interesting to investigate how *GAPDH* could contribute with FDG uptake level and tumor stage in building a composite prognostic marker, possibly also correlating it with the status of known NSCLC oncogenic genes (*PI3K*, *EGFR*, *KRAS*, *ALK*, etc.).

Finally, not only our results warn researchers from using *GAPDH* as housekeeper gene in NSCLC prognostic studies involving RQ-PCR measurements; we also suggest that any past NSCLC prognostic study using *GAPDH* as housekeeper gene should be considered potentially biased.

In conclusion, *GAPDH* gene expression level in resected tumor, as assessed by RQ-PCR or microarray, is an important prognostic factor in NSCLC, that confirms the importance of investigating metabolism in lung cancer.

## Competing interest

The authors declare that they have no competing interests.

## Authors’ contributions

RP, GS conceived the study, contributed to the interpretation of the data and wrote the manuscript. GS and AE performed RQ-PCR analysis and interpretation. RP performed all statistical analyses and public microarray dataset selection, collection and handling. SS and MT performed tumor sample handling, staging, storage and selection. MGDB, GB, ER, CG, CS fulfilled ethical authorizations, collected and stored patient data, clinical data and follow-up. UP, FG contributed to the interpretation of the data and revised manuscript. All authors reviewed and approved the manuscript.

## Supplementary Material

Additional file 1 ***GAPDH *primer for RQ-PCR and RQ-PCR data.** Primer sequence used for *GAPDH* RQ-PCR and RQ-PCR data for IST patients. Click here for file

Additional file 2 ***GAPDH *HR variation in low survival subsets.***GAPDH* Hazard Ratio (HR) variation in low cumulative survival subsets, investigated by random sampling from Shedden et al. 2008 dataset. Click here for file
