# Supplementary material for: Glyceraldehyde-3-phosphate dehydrogenase gene over expression correlates with poor prognosis in non small cell lung cancer patients
Source: Mol Cancer. 2013 Aug 29;12:97. doi: 10.1186/1476-4598-12-97 (PMC3766010; doi:10.1186/1476-4598-12-97)
Supplement: Additional file 1 — GAPDH primer for RQ-PCR and RQ-PCR data. Primer sequence used for GAPDH RQ-PCR and RQ-PCR data for IST patients. [file 1476-4598-12-97-S1.doc]

**Puzone at al 2013 - Additional File 1**

**Polymerase Chain Reaction primer sequences.**

GAPDH - Glyceraldeyde-3-phosphate-dehydrogenase

NM_002046 For GAAGGTGAAGGTCGGAGT lght:154

Rev CATGGGTGGAATCATATTGGA A

B2M - Beta-2-Microglobulin NM_004048 For TGACTTTGTCACAGCCCAAG lgth:113

Rev AGCAAGCAAGCAGAATTTGG

GUSB - Beta-Glucuronidase NM_000181 For GCC TGTGACCTTTGTGAGC lgth:109

Rev GTGCCCGTAGTCGTGATCC

PTINI DATA_ACC SEX AGE SMOK ISTO GRA TNM dead survty gapdh

05-D-06372 T-N 12/01/05 M 52 1 3 1 1 0 5.08 0.53

05-I-03890 M-G 07/15/05 M 82 1 2 3 1 1 3.58 1.12

05-I-04527 P-A 09/02/05 M 67 1 2 2 1 1 1.86 0.78

05-I-05053 M-A 09/30/05 F 68 0 1 2 1 1 3.73 0.46

05-I-05334 P-G 10/13/05 M 72 1 1 2 1 0 5.22 0.48

05-I-05653 B-O 10/28/05 M 57 1 3 1 3 0 5.18 0.8

05-I-06100 I-D 11/18/05 M 67 1 2 2 3 1 1.04 0.85

05-I-06395 B-R 12/02/05 M 58 1 1 3 1 1 3.32 0.2

05-I-06647 M-A 12/15/05 F 57 0 1 2 2 1 2.06 0.47

06-D-01053 P-D 02/16/06 M 69 0 1 2 2 1 2.33 1.95

06-D-01065 S-I 02/17/06 M 78 0 2 2 3 0 4.87 2.11

06-D-01086 G-M 02/17/06 M 59 1 1 2 1 1 2.06 0.85

06-D-01219 C-R 02/23/06 F 75 1 3 1 1 1 3.88 0.49

06-D-01374 L-L 03/02/06 F 74 1 1 3 2 0 4.83 3.87

06-D-01516 T-G 03/09/06 M 78 0 1 1 1 1 3.02 0.78

06-D-01541 M-E 03/09/06 F 70 0 3 1 1 0 4.81 0.16

06-D-01547 A-G 03/10/06 M 57 1 1 2 1 0 4.81 0.86

06-D-01574 C-V 03/10/06 M 49 1 3 1 3 0 4.81 0.55

06-D-01909 P-P 03/24/06 M 57 0 1 2 2 0 4.77 1.33

06-D-01940 B-G 03/27/06 M 68 1 2 2 1 0 4.76 2.19

06-D-02132 B-D 03/31/06 M 78 1 2 2 1 0 4.75 0.43

06-D-02285 B-M 04/06/06 F 57 0 3 1 1 0 4.74 0.66

06-D-02583 G-S 04/20/06 F 50 1 4 3 2 1 0.56 3.04

06-D-02685 S-A 04/27/06 M 77 0 3 1 1 0 4.68 0.4

06-D-02847 D-M 05/05/06 M 72 1 3 1 3 1 2.08 1.03

06-D-02871 P-G 05/05/06 M 59 1 3 1 1 0 4.66 0.2

06-D-03006 M-I 05/11/06 F 66 0 1 2 1 0 4.64 2.34

06-D-03248 G-M 05/19/06 M 80 1 4 3 1 0 4.62 1.2

06-D-03440 P-M 05/26/06 F 68 0 3 1 1 0 4.6 0.64

06-D-03578 Z-D 06/01/06 M 76 1 4 3 2 0 4.58 4.43

06-D-03901 F-F 06/15/06 M 66 0 2 2 1 0 4.55 1.62

06-D-04825 R-P 07/27/06 M 68 1 2 2 1 1 1.91 4.21

06-D-04874 G-G 07/28/06 M 62 1 1 2 3 1 3.33 2.49

06-D-04975 D-G 08/04/06 M 79 0 1 2 1 0 4.41 0.59

06-D-05046 R-G 08/09/06 M 69 1 2 2 2 0 4.39 1.68

06-D-05074 P-G 08/10/06 M 60 0 1 2 1 0 4.39 1.29

06-D-05463 C-G 09/07/06 M 56 1 1 3 3 0 4.32 2.49

06-D-05487 P-W 09/08/06 M 58 0 2 2 1 0 4.31 3.52

06-D-05503 C-L 09/08/06 M 69 1 1 1 2 0 4.31 0.7

06-D-05600 F-F 09/14/06 M 75 0 1 1 1 0 4.3 1.19

06-D-05778 F-G 09/22/06 M 66 1 1 2 3 1 1.25 5.86

06-D-05933 B-U 09/28/06 M 75 1 2 3 3 1 0.39 1.22

06-D-05969 D-L 09/29/06 F 75 1 1 2 2 1 2.69 1.16

06-D-06077 S-G 10/05/06 M 77 1 2 2 2 1 3.99 6.7

06-D-06120 M-U 10/06/06 M 79 0 1 2 1 1 4.24 2.49

06-D-07164 G-L 11/17/06 M 74 1 3 1 1 0 4.12 0.34

06-D-07370 F-G 11/24/06 F 59 0 1 3 1 1 0.32 1.76

06-D-07572 C-G 12/01/06 F 75 1 1 3 2 0 4.08 1.82

06-D-07902 C-G 12/15/06 M 78 1 2 2 3 1 0.12 3.31

06-I-00380 V-P 01/18/06 F 71 1 1 2 3 1 1.39 0.79

06-I-00545 M-E 01/26/06 F 72 0 2 3 1 0 4.93 1.71

06-I-00881 B-F 02/09/06 M 48 0 2 3 3 0 4.89 2.3

06-I-01327 B-A 03/01/06 F 75 1 2 2 1 0 4.84 0.46

06-I-02300 Z-G 04/07/06 M 79 1 2 2 1 0 4.73 3.26

06-I-03744 P-C 06/08/06 M 58 1 1 3 2 1 1.58 0.91

06-I-03757 L-M 06/09/06 M 50 1 1 3 3 1 1.4 1.21

06-I-03935 R-N 06/16/06 M 70 0 1 1 1 0 4.54 0.57

06-I-04041 S-C 06/21/06 F 47 1 1 2 3 1 0.77 0.88

06-I-04205 U-R 06/28/06 F 72 1 1 3 3 1 2.25 1.71

06-I-04231 L-B 06/29/06 M 56 1 2 2 1 0 4.51 0.86

06-I-04409 P-E 07/07/06 M 77 0 1 2 3 1 1.48 1.38

06-I-04549 C-G 07/14/06 M 70 1 1 2 3 1 1.99 3.09

06-I-04725 B-F 07/21/06 M 61 1 2 2 2 1 1.43 3.01

06-I-05052 B-G 08/09/06 M 78 1 1 2 3 1 0.97 2.28

06-I-05057 L-G 08/10/06 M 68 0 2 2 3 1 0.66 1.67

06-I-05103 L-P 08/16/06 M 54 0 2 2 1 0 4.38 1.97

06-I-05113 C-A 08/16/06 M 74 1 1 2 1 1 1.93 4.96

06-I-05172 M-A 08/22/06 M 63 1 2 2 1 0 4.36 1.35

06-I-06411 B-T 10/19/06 M 64 1 1 2 3 1 1.8 1.55

06-I-06443 P-N 10/20/06 M 76 0 2 2 1 0 4.2 1.11

06-I-06779 B-A 11/02/06 F 75 1 3 2 1 0 4.16 0.35

06-I-07334 B-N 11/23/06 M 65 1 2 2 1 0 4.1 0.45

06-I-07552 P-B 11/30/06 M 77 1 5 3 2 1 0.53 1.17

06-I-07739 S-A 12/07/06 M 74 0 2 3 2 1 2.56 0.68

06-I-08082 V-F 12/22/06 M 58 1 2 2 3 1 1.3 3.08

07-D-00067 C-E 01/04/07 M 71 1 1 2 1 0 3.99 1.03

07-D-00072 S-V 01/05/07 M 63 1 1 2 1 0 3.99 0.92

07-D-00613 S-G 01/26/07 M 74 1 2 2 1 1 0.18 2.4

07-D-00763 C-T 02/01/07 F 52 0 1 2 3 0 3.91 0.67

07-D-01587 S-C 03/02/07 M 51 0 3 2 3 0 3.83 0.74

07-I-01152 G-C 02/15/07 F 73 1 3 1 1 0 3.87 0.48

07-I-01216 B-R 02/16/07 M 73 1 2 2 1 0 3.87 5.13

Sample R code . (data loaded as Bioconductor's expression set object)

age<-eset$AGE; TNM<-as.factor(eset$TNM); tsur<-(eset$survty); stat<-eset$dead;

str(stat); str(age); str(TNM); str(tsur);

library(survival);

fmla <- as.formula("Surv(tsur,stat)~ exprs(eset)[\"gapdh\",] +TNM"); fit<-coxph(fmla); fit; conf<-confint(fit);

fmla <- as.formula("Surv(tsur,stat)~ exprs(eset)[\"gapdh\",]"); fit<-coxph(fmla); fit; conf<-confint(fit);

so<-summary(survfit(Surv(tsur,stat)~1)); last<-length(so$surv); so$surv[last]; so$lo[last]; so$up[last];

gmed<-median(as.matrix(exprs(eset["gapdh",])),na.rm = TRUE);gfact<-as.factor(exprs(eset["gapdh",])>gmed);

plot(survfit(Surv(tsur,stat)~gfact),col=c(1,2), xlim=c(0,5), main="KM surv by GAPDH", ylab="Surv", xlab="years");
