# Supplementary material for: Glyceraldehyde-3-phosphate dehydrogenase gene over expression correlates with poor prognosis in non small cell lung cancer patients
Source: Mol Cancer. 2013 Aug 29;12:97. doi: 10.1186/1476-4598-12-97 (PMC3766010; doi:10.1186/1476-4598-12-97)
Supplement: Additional file 2 — GAPDH HR variation in low survival subsets.GAPDH Hazard Ratio (HR) variation in low cumulative survival subsets, investigated by random sampling from Shedden et al. 2008 dataset. [file 1476-4598-12-97-S2.doc]

Puzone et al 2013 Additional file 2 - ***GAPDH* HR do not change in low cumulative survival subsets**

**Analysis of *GAPDH* Hazard Ratio (HR) in subsets of Shedden et al 2008 obtained by random sampling from not-adjuvant-treated patients.**

By investigating Shedden et al, 2008 (Sh2008) microarray dataset[1], we found that in the adjuvant treated only patient subset, the 5-years cumulative survival value was much lower than in the not adjuvant-treated patient subset; this was probably due to the fact that clinicians selected the predictably worse prognosis patients to be referred to adjuvant treatments. However, we wanted to investigate if selecting a worse prognosis patient subset was, per se, much affecting *GAPDH* HR calculation in the subset. To address at least in part this issue, we generated 10000 100-patient subsets by random sampling from the 330 not adjuvant-treated patients in Sh2008, and calculated *GAPDH* HR and cumulative survival for each subset by using survival Cox regression models. In appendix are reported similar results obtained by shaping the original set in random subsamples built to feature low survivals, and some sample R code.


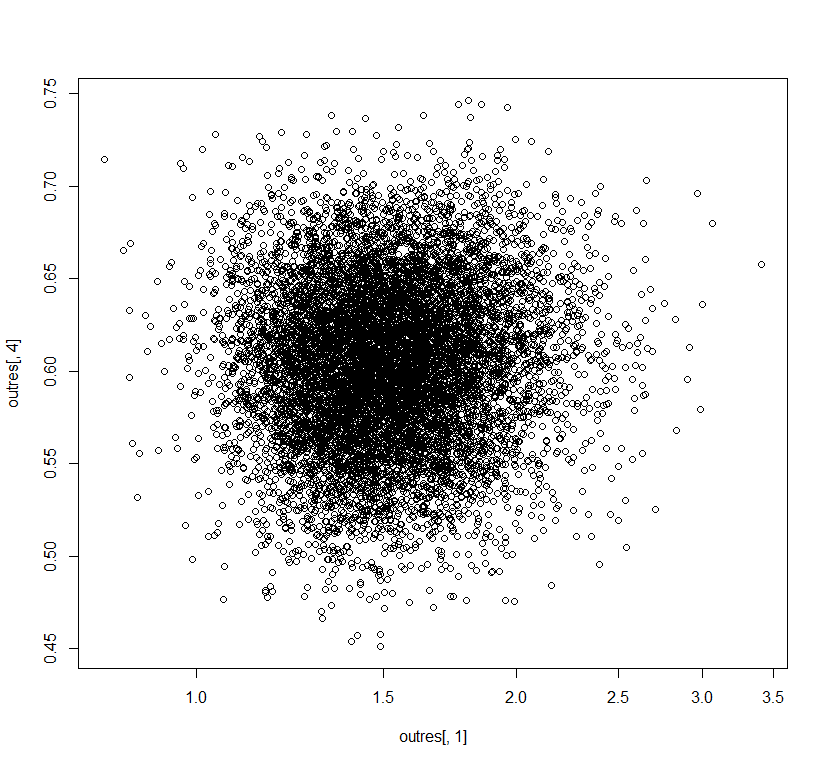


Fig.S1. X axes are *GAPDH* HRs; Y axes are subsets' 5-years cumulative survival (surv). Each point represents the survival Cox regression model result for a single 100 patient subset; 10000 subsets are plotted, each obtained by random sampling from Sh2008 not adjuvant-treated patients. Dataset's cumulative survival correlation with *GAPDH* HR is negligible (Pearson's r= 0.05 p<.0001). Calculations and plots were done by using R 2.14 statistical software.

1. Shedden K et al: Gene expression-based survival prediction in lung adenocarcinoma: a multi-site, blinded validation study. Nat Med 2008, 14: 822-827.

Appendix 1


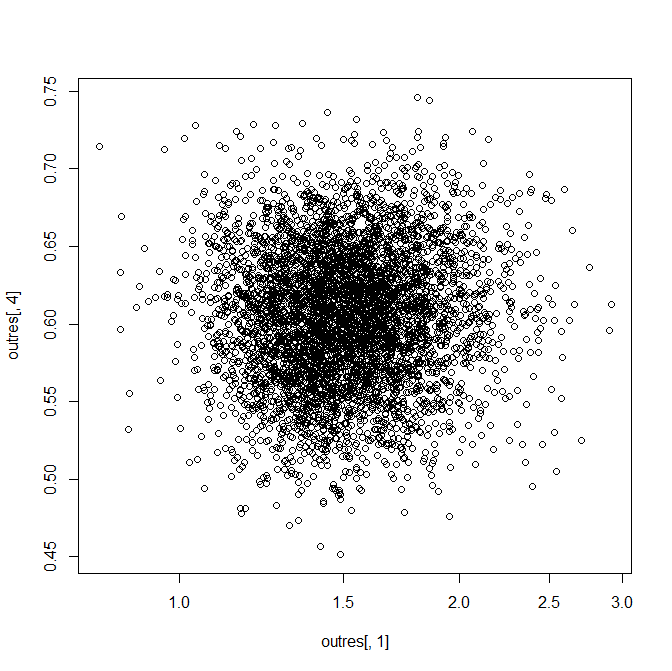


330 untreated patients

KM surv 5y value and CI:

[1] 0.6038352

[1] 0.5502679

[1] 0.6626172

Pearson's product-moment correlation

data: exp(outres[, 1]) and outres[, 4]

t = 7.2365, df = 9998, p-value = 4.943e-13

alternative hypothesis: true correlation is not equal to 0

95 percent confidence interval:

0.05265796 0.09165393

sample estimates:

cor

0.07218353


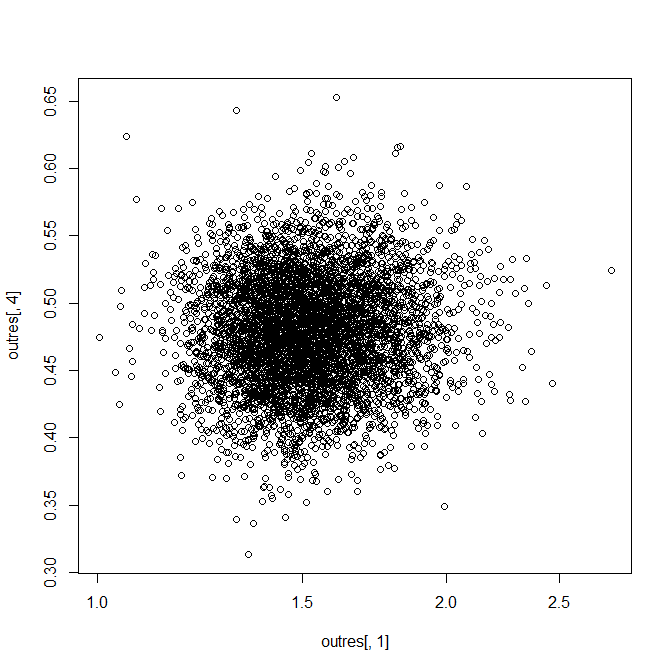
224 patients (330 untreated without 50% of the patients with surv>3 years)

KM surv 5y value and CI:

[1] 0.4783678

[1] 0.4124571

[1] 0.554811

Pearson's product-moment correlation

data: exp(outres[, 1]) and outres[, 4]

t = 3.993, df = 4998, p-value = 6.618e-05

alternative hypothesis: true correlation is not equal to 0

95 percent confidence interval:

0.02871668 0.08397910

sample estimates:

cor

0.05639108


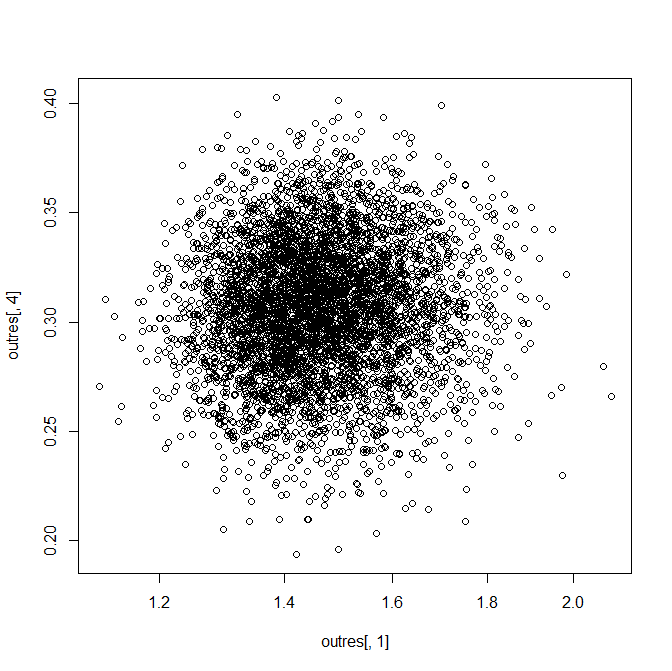
154 patients (330 untreted without 75% of the patients with surv>3years)

KM surv 5y value and CI:

[1] 0.306292

[1] 0.2351474

[1] 0.3989617

Pearson's product-moment correlation

data: exp(outres[, 1]) and outres[, 4]

t = 3.0589, df = 4998, p-value = 0.002233

alternative hypothesis: true correlation is not equal to 0

95 percent confidence interval:

0.01552679 0.07086186

sample estimates:

cor

0.04322748

Sample R code

#---------sampling test low1gceset is Sh2008 Bioconductor dataset object

library(survival)

#lowgceset<-low1gceset[,low1gceset$ADJUVANT_CHEMO=="No" & low1gceset$ADJUVANT_RT=="No"]; dim(lowgceset);

#lowgceset<-low1gceset;

tsur<-tsur0<-as.numeric(as.character(lowgceset$survty)); stat<-stat0<-as.numeric(as.character(lowgceset$dead)); stat[tsur>5]=0; tsur[tsur>5]=5; age<-as.numeric(as.character(low1gceset$AGE));

so<-summary(survfit(Surv(tsur,stat)~1)); last<-length(so$surv); so$surv[last]; so$lo[last];so$up[last];

set.seed(1234); testn=5000; samp=100; outres<-matrix(0, testn, 6); patzn<-dim(lowgceset)[2];

for(i in 1:testn) { low1wkk<-lowgceset[,sample(patzn,samp)]

tsur<-as.numeric(as.character(low1wkk$survty)); stat<-as.numeric(as.character(low1wkk$dead));stat[tsur>5]=0; tsur[tsur>5]=5;

age<-as.numeric(as.character(low1wkk$AGE));

fmla <- as.formula("Surv(tsur,stat)~ exprs(low1wkk)[\"GAPDH\",]+age"); fit<-coxph(fmla); s<-summary(fit); conf<-confint(fit);

outres[i,1]<-s$coe[1,2];outres[i,2]<-exp(conf)[1,1]; outres[i,3]<-exp(conf)[1,2];

so<-summary(survfit(Surv(tsur,stat)~1)); last<-length(so$surv); outres[i,4]<-so$surv[last]; outres[i,5]<-so$lo[last];outres[i,6]<-so$up[last];

}

plot(outres[,1],outres[,4], log="x"); cor.test(exp(outres[,1]),outres[,4])

# shaper to get a random lower survival subset

scut<-2 # to exclude 1/scut fraction of longsurv (>3 years surv) patients from the adjuvant naïve set

lowgceset<-low1gceset[,low1gceset$ADJUVANT_CHEMO=="No" & low1gceset$ADJUVANT_RT=="No"]; dim(lowgceset);

tsur<-tsur0<-as.numeric(as.character(lowgceset$survty)); tsur[tsur>5]=5;

loset<-tsur<3; hiset<-tsur>=3; set.seed(12345); hisetcut<-sample(length(hiset),length(hiset)/scut); hiset[hisetcut]<-FALSE; lowgceset<-lowgceset[,(loset | hiset)];dim (lowgceset);

tsur<-as.numeric(as.character(lowgceset$survty)); stat<-as.numeric(as.character(lowgceset$dead));stat[tsur>5]=0; tsur[tsur>5]=5;

so<-summary(survfit(Surv(tsur,stat)~1)); last<-length(so$surv); so$surv[last]; so$lo[last];so$up[last];
